# pH-sensitive dual drug loaded janus nanoparticles by oral delivery for multimodal analgesia

**DOI:** 10.1186/s12951-021-00974-6

**Published:** 2021-08-06

**Authors:** Lin Liu, Wendong Yao, Xiaowei Xie, Jianqing Gao, Xiaoyang Lu

**Affiliations:** 1 Department of Clinical Pharmacy, The First Affiliated Hospital, Zhejiang University School of Medicine, 79 Qingchun Road, Hangzhou, 310003 PR China; 2 Institute of Pharmaceutics, College of Pharmaceutical Sciences, Zhejiang University, 866 Yuhangtang Road, Hangzhou, 310058 PR China; 3 Department of Pharmacy, The First Affiliated Hospital of Zhejiang Chinese Medical University, 310018 Hangzhou, PR China; 4 College of Pharmaceutical Sciences, Zhejiang Chinese Medical University, 310053 Hangzhou, PR China

**Keywords:** Janus nanoparticles, pH-sensitive, Peptide drugs, Oral delivery, Multimodal analgesia

## Abstract

**Background:**

Based on the concept of “multimodal analgesia”, a novel dual drug delivery system was designed to achieve synergistic analgesia between najanajaatra venom protein (αCT) and resveratrol (Res). In order to meet the joint loading of two drugs with different physicochemical properties without affecting each other, an oral Janus nanoparticle (JNP) with a unique cavity structure and synergistic drug delivery was constructed using an improved double emulsion solvent evaporation method, and combined with low-molecular-weight chitosan/sodium alginate and PLGA to achieve its pH-responsive.

**Results:**

The synthesized αCT/Res-JNPs are homogeneous in shape, with a two-compartment structure, approximately 230 nm in size, and zeta potential of 23.6 mV. Drug release assayed in vitro show that JNP was stable in simulated gastric juice (pH = 1.2) but was released in phosphate buffer saline (pH = 7.4). After intragastric administration in rats, PK evaluation showed that αCT/Res-JNPs could significantly improve the oral bioavailability, and the simultaneous encapsulation of the two drugs had no significant interaction on PK parameters. An obvious synergistic analgesic effects of αCT/Res-JNPs was confirmed in a spinal cord injury and acute pain model. Confocal laser scanning microscopy and single-pass intestinal perfusion model provided strong evidence that αCT/Res-JNPs could pass through intestinal epithelial cells, and the endocytosis pathway was mainly involved in the mediation and pinocytosis of reticulin. The concentrations of αCT and Res from αCT/Res-JNP in lymphatic transport were only about 8.72% and 6.08% of their blood concentrations at 1 h, respectively, which indicated that lymphatic transport in the form of JNP has limited advantages in improving the oral bioavailability of Res and αCT. Cellular uptake efficiency at 4 h was about 10–15% in Caco-2 cell lines for αCT/Res-JNP, but was reduced to 7% in Caco-2/HT29-MTX co-culture models due to the hindrance by the mucus layers. Approximately 12–17% of αCT/Res-JNP were transported across Caco-2/HT29-MTX/Raji monolayers. The cumulative absorption of JNP in three cell models was higher than that of free drug.

**Conclusions:**

This study investigated the contribution of Janus nanoparticles in oral absorption, and provide a new perspective for oral administration and analgesic treatment of dual drug delivery system containing peptide drugs.

**Graphic Abstract:**

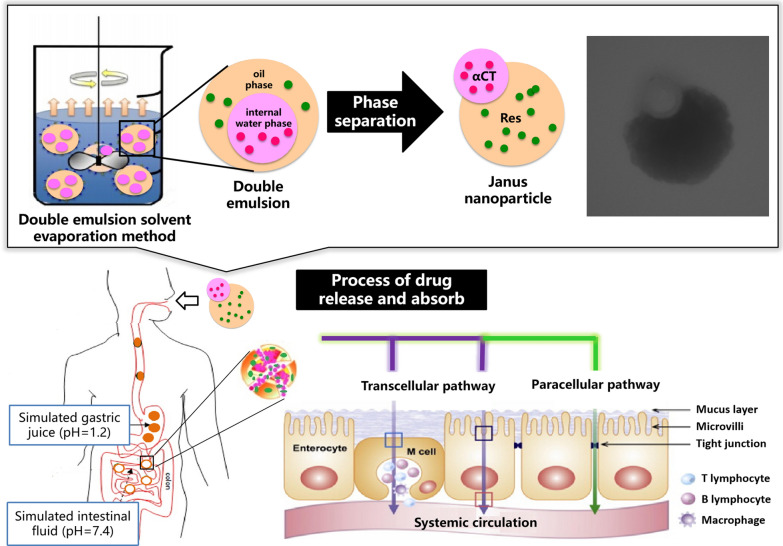

**Supplementary Information:**

The online version contains supplementary material available at 10.1186/s12951-021-00974-6.

## Background

In clinical practice, a single drug or method cannot achieve complete pain relief [1]. Therefore, the concept of “multimodal analgesia” has been proposed, that is, the combination of analgesic drugs and/or measures with different mechanisms of action to achieve a synergistic analgesic effect, reducing the dose of each drug and its corresponding side effects [2], which represents the main development direction of clinical analgesia technology. For instance, a combination of multiple drugs to inhibit central pain signals can achieve a compound central nerve block through the accumulation or synergy of central pain analgesia. α-Cobrotoxin (αCT), an important component of the najanajaatra venom protein, is composed of 62 amino acids and 4 disulfide bonds. It has a strong central analgesic effect, that acts mainly by inhibiting the release of acetylcholine [3]. Its analgesic potency is much higher than that of morphine and it has no addictive effect, however, its effect is slow. It has been approved for the treatment of chronic pain such as intractable neuralgia and cancer pain [4]. Resveratrol (Res) is a natural stilbene compound extracted from plants, It has been shown to have a definite central analgesic effect, [5–7] which is quick but transient. Based on the characteristics of the two drugs, a combination of Res and αCT can compensate for the inherent defects of both drugs and play a central synergistic analgesic effect, [8] that has also been verified in our preliminary experiment (Additional file 1: S1). In order to avoid multiple administration, we continue to investigate the feasibility of these two drugs in a common carrier for synergistic analgesia.

Among the routes of administration of combined analgesics, the oral route is another research hotspot that many medical workers are working towards. Debanjan et al. [9] have confirmed that the peptide analgesic dalagan can achieve intracerebral delivery and central analgesia through the oral route. However, it is well known that the oral administration of peptide drugs such as αCT faces three major obstacles in the gastrointestinal environment: acid hydrolysis [10], enzyme degradation [11], and low permeability caused by the mucosal barrier [12]; therefore, it is difficult to take orally, which is the major drawback in the clinical use of peptide drugs. On the other hand, Res is a small molecule that is insoluble in water. Although it has high membrane permeability, its plasma half-life is very short (approximately 8–14 min) [13]. Due to its rapid and extensive metabolism in vivo, the oral bioavailability of Res is almost zero in both humans and animals [14, 15].

To solve the above difficulties, a novel construction approach was explored for an oral dual drug-loaded particle delivery system that can meet the joint loading of two drugs with different physicochemical properties without affecting each other. The key to this approach was to accurately control the internal structure of the particles. Many studies have reported the application of Janus particles in drug delivery systems [16–18]. In contrast to ordinary particles, Janus particles show unique advantages in that it is possible to precisely control their internal cavity structures, owing to their anisotropy and dual functions. Their two-compartment structures enable the encapsulation of drugs with different properties (such as hydrophilicity and hydrophobicity), subsequently achieving independent release and delivery, thus avoiding various pharmacokinetic problems of single-structure double-drug-loaded particles, which provides a solid foundation for its application in double drug delivery systems [19]. Therefore, in this study, Janus nanoparticle (JNP) was constructed to realize the independent encapsulation of both αCT and Res (αCT/Res-JNP). Meanwhile, the carrier materials were further optimized to ensure the stable delivery of αCT and Res in the gastrointestinal environment and improve the oral bioavailability. The results will help evaluate whether the unique cavity structure, synergistic delivery, and environmental sensitivity of JNP can improve the oral bioavailability of both αCT and Res, and provide a new perspective for the oral delivery and analgesic treatment of dual drug delivery systems containing peptide drugs.

## Results

### Preparation and characterization of αCT/Res-JNPs

**Fig. 1:**
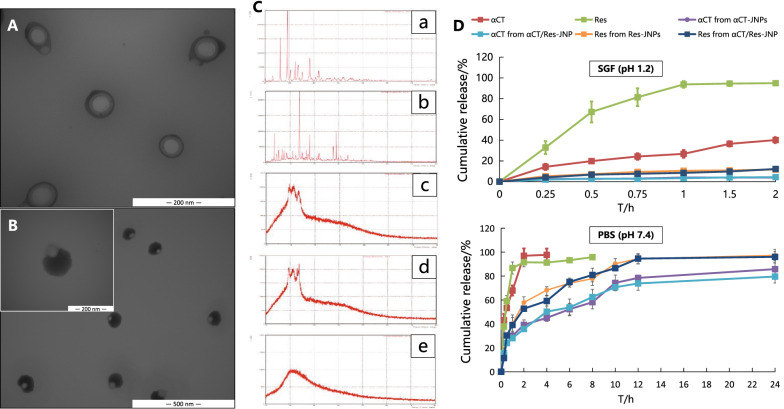
Characterization and cumulative in vitro release profiles of Janus nanoparticles. TEM image showing the structure transformation of αCT/Res-JNP under different volume ratio of surfactant to W1/O emulsion: **A** Core–shell structure (Surfactant: W1/O emulsion = 1:4), **B** Dumbbell-shaped with asymmetric attached nodes (Surfactant: W1/O emulsion = 5:12); **C** XRD spectra of (a) αCT, (b) Res, (c) PLGA:LWMC:ALG mixture (1:1:1), (d) αCT:Res:PLGA:LWMC:ALG mixture (1:1:1:1:1), (e) αCT/Res-JNP. **D** Cumulative in vitro αCT and Res release profiles of JNP in SGF (pH 1.2) and PBS (pH 7.4) (n = 3, mean ± SD)

αCT/Res-JNPs were successfully prepared by an improved double emulsion solvent evaporation method. As seen from Fig. 1, the particle size of αCT/Res-JNPs ranged from 283.2 nm to 350.8 nm, with a PDI of 0.196 to 0.248, and a Zeta potential of approximately 23.6 mv. The morphology of the αCT/Res-JNPs was dumbbell-shaped with asymmetric attached nodes (Fig. 1B), which was also found in many previous reports [19]. The final formation of JNP structure was affected by the amount of surfactant (PVA:SDBS = 1:3). When the volume ratio of surfactant to W1/O emulsion was increased from 1/4 to 5/12, the droplet transformed from the core–shell structure (Fig. 1A) into Janus structure (Fig. 1B). X-ray diffraction (XRD) was used to further verify the structure of αCT/Res-JNPs, and it was found that compared with the drug/polymer physical mixture, the derivative peaks of αCT and Res disappeared in the XRD spectra of αCT/Res-JNP lyophilized powder (Fig. 1C), suggesting that αCT and Res have been encapsulated in JNP. The entrapment efficiency (EE%) and drug loading (DL%) of αCT were 58.14 ± 2.36% and 7.93 ± 0.32%, respectively, whereas those of Res were 87.49 ± 3.43% and 59.28 ± 2.32%, respectively.

### In vitro release behavior of αCT and Res from αCT/Res-JNP

Due to the rapid degradation of peptides in simulated gastrointestinal juice containing enzymes, the solvent without enzymes was chosen for the in vitro drug release study. We measured the release behavior of αCT/Res-JNP in simulated gastric juice (SGF, pH 1.2) for 2 h and phosphate buffer saline (PBS, pH 7.4) for 24 h to simulate the pH environment of the fasting stomach and small intestine, respectively. Because Res is insoluble in water, 2% sodium dodecyl sulfate (w/v) was added to the release medium to increase its dissolution. As shown in Fig. 1D, the cumulative release of the αCT solution in SGF was approximately 40.17%. It was speculated that free αCT was affected by acid hydrolysis to a certain extent, while the cumulative release of αCT encapsulated in αCT/Res-JNP was almost undetectable. Similarly, the release rate of free Res was 93.76% within 1 h, while the release rate of Res encapsulated in αCT/Res-JNP was not obvious, indicating that the encapsulation of JNP had a certain resistance to free αCT and Res in an acidic environment.

The release rates of αCT and Res in PBS were 67.89% and 86.71%, respectively. αCT was released in approximately 30.03% and 28.11% of αCT-JNP and αCT/Res-JNP, respectively, while Res was released in approximately 40.21% and 39.09% of Res-JNP and αCT/Res-JNP, respectively. Compared with free αCT and Res, the JNPs have obvious sustained-release characteristics. Within 12 h, the cumulative release of αCT in αCT-JNP and αCT/Res-JNP was 78.44% and 73.82%, respectively, while the cumulative release of Res in Res-JNP and αCT/Res-JNP was 94.41% and 94.57%, respectively. Moreover, the results showed that there was no significant interaction between αCT and Res in JNP.

### In vitro stability

**Fig. 2:**
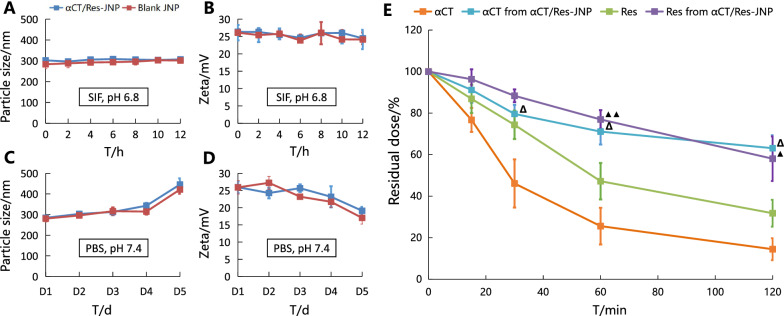
In vitro stability of αCT/Res-JNP in different media (n = 3, mean ± SD). **A** Particle size (SIF, pH 6.8), **B** Zeta potential (SIF, pH 6.8), **C** Particle size (PBS, pH 7.4), **D** Zeta potential (PBS, pH 7.4), and **E** the residual drug content of JNP (∆: Comparison of free αCT and residual αCT in αCT/Res-JNP, *p* < 0.05; ▲: Comparison of free Res and residual Res in αCT/Res-JNP, *p* < 0.05; ▲▲: Comparison of free Res and residual Res in αCT/Res-JNP, *p* < 0.01)

The stability of αCT/Res-JNP in various simulated physiological media is a prerequisite for the evaluation of their integrity in vivo. JNP maintained fair particle size and zeta potential stability in simulated intestinal fluid (SIF, pH 6.8) for 12 h (Fig. 2A, B). However, significant changes in particle size and zeta potential were observed in PBS (pH 7.4). The particle size of αCT/Res-JNP increased significantly on D5 (1.6 times larger than that on D1) (Fig. 2C) and its zeta potential decreased gradually with time (Fig. 2D).

The concentrations of αCT and Res from αCT/Res-JNP in SIF containing 0.2% trypsin were determined to evaluate the protective effect of JNP on αCT and Res. We used trypsin to investigate the resistance of αCT/Res-JNP to digestive enzymes. In the first hour, free αCT and free Res remained at 25.60% and 47.23%, respectively, indicating that both drugs were rapidly degraded in the presence of trypsin, while the residual amount of αCT and Res only decreased to 71.11 ± 6.23% and 76.96 ± 4.48%, respectively, in αCT/Res-JNP. Within 2 h, the residual amount of αCT and Res decreased to 63.12 ± 6.24% and 58.06 ± 10.74%, respectively, in αCT/Res-JNP (Fig. 2E). It can be seen that compared with free αCT or free Res, αCT/Res-JNP can protect drugs against trypsin degradation and increase their stability in a simulated intestinal environment containing trypsin.

### Pharmacokinetics study

**Fig. 3:**
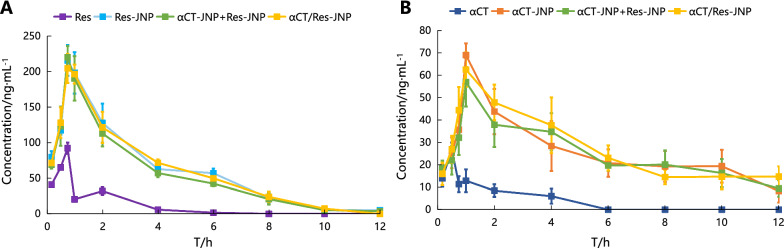
Plasma drug level profiles after intragastric administration in rats (n = 5, mean ± SD). **A** Drug concentration–time curve of Res, **B** Drug concentration–time curve of αCT

The blood concentrations of αCT or Res in rats at different times after intragastric administration was determined using PKSolver software. Figure 3A showed that Res was rapidly absorbed in the gastrointestinal tract of rats. The peak concentration of Res was 92.06 ± 8.00 ng/mL at 10 min after intragastric administration. The t_1/2_ of αCT was at 0.17 ± 0.03 h after intragastric administration, indicating that αCT was rapidly absorbed and reached its peak concentration in about 30 min, but its concentration in plasma was relatively low, with a peak concentration of 26.85 ± 3.00 ng/mL (Fig. 3B). Compared with the Res group or αCT group, t_1/2_ α, t_1/2_ β, AUC_0-t_, AUMC_t_, and MRT_t_ were increased in the various forms of JNP groups (p < 0.01, or p < 0.05) (Tables 1 and 2), which suggested that the oral bioavailability of both drugs was significantly improved. Meanwhile, JNP co-loaded with αCT and Res did not affect the PK behavior of either drug compared with the loading of a single drug only.

**Table 1:** Main pharmacokinetic parameters of Res in each group (n = 5, mean ± SD)

Parameters	Unit	Parameter value			
		Free Res	Res-JNP	αCT-JNP + Res-JNP	αCT/Res-JNP
t1/2α	1/h	0.19 ± 0.02	1.25 ± 0.02**	1.11 ± 0.36**	1.47 ± 0.22**
t1/2β	h	1.09 ± 0.07	4.65 ± 0.94**	4.76 ± 1.08**	4.35 ± 1.37**
Cmax	ng/ml	92.06 ± 8.00	215.53 ± 21.64**	249.98 ± 15.93**	204.52 ± 20.53**
Tmax	h	0.17	0.75	0.75	0.75
AUCt	ng/ml*h	84.64 ± 3.80	750.91 ± 167.35**	606.16 ± 54.78**	677.96 ± 70.71**
AUMCt	ng/ml*h^2	118.18 ± 19.08	2171.83 ± 296.19**	1868.18 ± 177.18**	1964.63 ± 217.18**
MRTt	h	1.56 ± 0.52	3.73 ± 1.25**	4.26 ± 2.45*	3.01 ± 1.77

Compared with free Res, ^*^p < 0.05, ^**^ p < 0.01

**Table 2:** Main pharmacokinetic parameters of αCT in each group (n = 5, mean ± SD)

Parameters	Unit	Parameter value			
		Free αCT	αCT-JNP	αCT-JNP + Res-JNP	αCT/Res-JNP
t1/2α	1/h	0.17 ± 0.03	0.36 ± 0.02**	0.34 ± 0.04**	0.35 ± 0.03**
t1/2β	h	2.69 ± 0.36	5.61 ± 0.43**	5.37 ± 0.31**	5.40 ± 0.17**
Cmax	ng/ml	26.85 ± 3.00	68.97 ± 5.27**	56.92 ± 10.91**	62.50 ± 7.72**
Tmax	h	0.5	1.0	1.0	1.0
AUCt	ng/ml*h	67.65 ± 17.91	168.17 ± 18.36**	209.84 ± 28.92**	230.71 ± 36.01**
AUMCt	ng/ml*h^2	112.44 ± 12.97	655.91 ± 42.06**	649.69 ± 115.07**	758.61 ± 82.74**
MRTt	h	1.91 ± 0.02	1.91 ± 0.02**	3.34 ± 0.05**	3.38 ± 0.06**

Compared with free αCT, ^**^ p < 0.01

### Analgesic effect

**Fig. 4:**
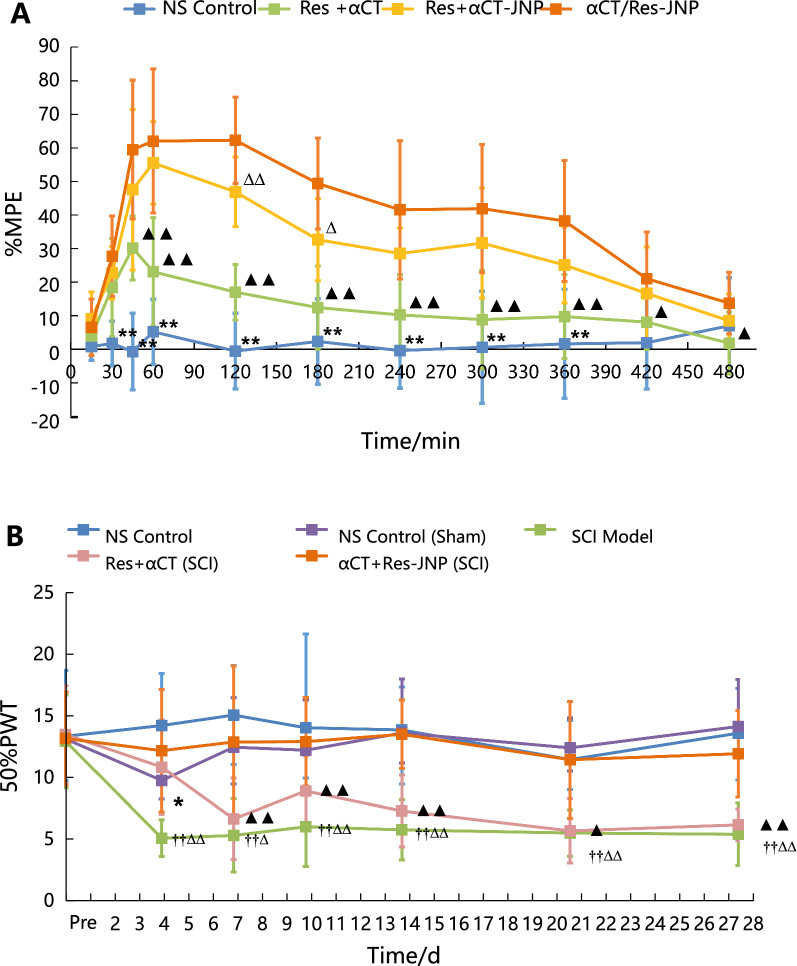
The pain threshold of rats after intragastric administration in rats. (n = 10, mean ± SD). **A** Maximal possible effect (%MPE) of hot plate test (*: NS Control *vs.* αCT/Res-JNP, *p* < 0.05; **: NS Control *vs.* αCT/Res-JNP, *p* < 0.01; ▲: Res + αCT *vs.* αCT/Res-JNP, *p* < 0.05; ▲▲: Res + αCT *vs.* αCT/Res-JNP, *p* < 0.01; ∆: Res + αCT-JNP *vs.* αCT/Res-JNP, *p* < 0.05; ∆∆: Res + αCT-JNP *vs.* αCT/Res-JNP, *p* < 0.01); **B** 50% paw withdrawal threshold (50% PWT) in rats with central pain after spinal cord injury (††: SCI model *vs.* NS control, *p* < 0.01; *: Sham operation *vs.* αCT/Res-JNP, *p* < 0.05; ∆: SCI model *vs.* αCT/Res-JNP, *p* < 0.05; ∆∆: SCI model *vs.* αCT/Res-JNP, *p* < 0.01; ▲: Res + αCT *vs.* αCT/Res-JNP, *p* < 0.05; ▲▲: Res + αCT *vs.* αCT/Res-JNP, *p* < 0.01)

It has been reported that a high dose of αCT (100 μg·kg^−1^) via intraperitoneal injection can significantly enhance pain sensitivity, while a low dose of αCT can improve the pain threshold and action ability in animal models [20]. The purpose of this study was to investigate the analgesic effect of the drugs, so low-dose αCT was selected (50 µg kg^−1^), and the dose of Res was set on this basis. The evaluation of the analgesic effect using the hot plate method is shown in Fig. 4A. The results showed that there was no significant difference in the basic pain threshold among the four groups (p > 0.05). The analgesic effect of all groups was compared with that of αCT/Res-JNP by using the percentage of the maximal possible effect (%MPE). The %MPE of αCT/Res-JNP was significantly higher than that of NS (p < 0.01, p < 0.05) and Res solution combined with αCT solution (45 min to 360 min, p < 0.01; 420 min, p < 0.05). Compared with the Res solution combined with αCT-JNP, the maximum analgesic efficiency of the combination was significantly increased after administration, and the difference was very significant at 120 min (p < 0.01), and significant at 180 min and 360 min (p < 0.05, respectively).

To investigate the analgesic effect of αCT/Res-JNP on central pain, the analgesic effect of αCT/Res-JNP in spinal cord injury (SCI) model rats was further investigated using the von Frey filament method. To avoid the early stage of spinal cord shock after SCI, the von Frey filament test was started from D4. The analgesic effects of each group are shown in Fig. 4B. The results showed that there was no significant difference in the preoperative pain threshold of the five groups (p > 0.05), while the paw retraction reflex threshold of SCI rats was significantly lower than that of normal rats and sham-operated rats. The acute stage of central pain after spinal cord injury appeared approximately 4 days after operation, and chronic neuropathic pain after spinal cord injury began to appear 10 days after operation. The pain symptoms then began to aggravate and lasted for a long time. The modeling results were consistent with those of other previous studies [21]. Compared with the αCT/Res-JNP group, the postoperative foot pain threshold of free αCT combined with the free Res group was significantly decreased. Compared with the sham-operated group, the pain threshold of the SCI group treated with αCT increased at D4 after the operation, and then decreased slightly, but still maintained a stable trend. There was no significant difference in the pain thresholds between the two groups (p > 0.05). Furthermore, there was no significant difference in the pain threshold between the SCI group treated with αCT/Res-JNP and the normal control group (p > 0.05). The above results showed that αCT/Res-JNP significantly reversed mechanical hyperalgesia and had significant analgesic effects against mechanical hyperalgesia, even during the formation of chronic central pain from D10 to D28.

### Cellular uptake and trans-monolayer transport

**Fig. 5:**
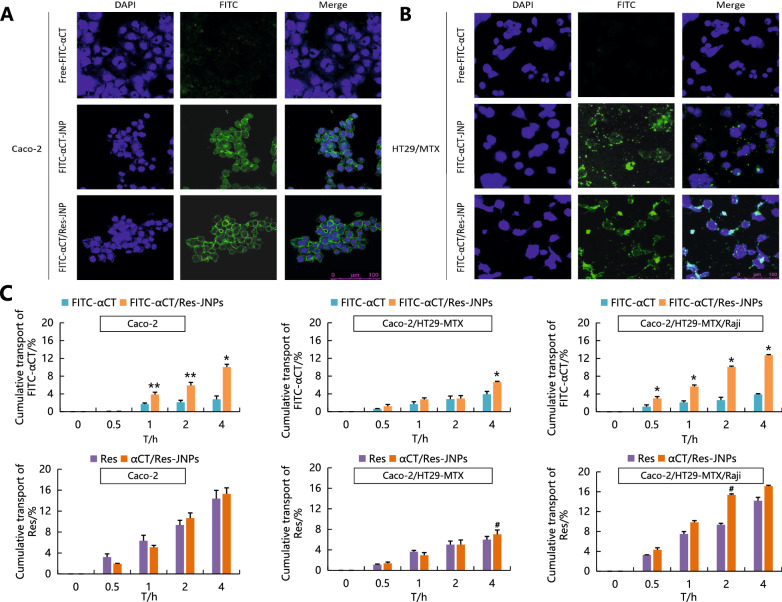
Cellular uptake of FITC-αCT by Caco-2 cells (**A**) and HT29/MTX cells (**B**) were observed by confocal laser scanning microscope. Green signal showed internalized FITC-αCT, and blue signal showed nucleus. **C** Cumulative trans-monolayer transport of JNP in Caco-2, Caco-2/HT29-MTX, and Caco-2/HT29-MTX/Raji monolayers (n = 3, ^**^: FITC-αCT vs. FITC-αCT/Res-JNP, p < 0.01; ^*^: FITC-αCT vs. FITC-αCT/Res-JNP, p < 0.05; ^#^: Rea vs. αCT/Res-JNPs, p < 0.05)

The study of cellular uptake and trans-monolayer transport were conducted to help understand the mechanisms of oral absorption of αCT/Res-JNP. The cytotoxicity test and its results are shown in Additional file 2: Figure S2, which suggested the experimental concentration of FITC-αCT-JNP and FITC-αCT/Res-JNP was not exceed 10% (i.e. αCT is about 0–4 μg/ml, Res is about 0–175 μg/ml). Figure 5 showed that FITC-αCT from JNP of both groups was distributed in Caco-2 cells and HT29/MTX cells, while free FITC-αCT showed no obvious green fluorescence, suggesting that JNP could be absorbed by Caco-2 and HT29/MTX cells. In addition, there was no significant difference in the cell uptake and fluorescence intensity of FITC-αCT in JNP with double drug loading compared to JNP with single drug loading, suggesting that double drug loading had no significant effect on the uptake of a single drug.

In order to elucidate the endocytosis pathway of JNPs, we used specific endocytosis inhibitors to block different endocytosis pathways, including chlorpromazine, which blocks the Trellin-mediated pathway by inhibiting Rho GTPase [22]; genistein, which blocks the caveolin-mediated pathway [23]; amiloride, which blocks the macropinocytosis pathway [24]; and nocodazole, which inhibits phagocytosis [25]. The uptake of JNPs by Caco-2 cells without endocytosis inhibitors was defined as the control group, that is, the uptake of JNP by Caco-2 cells in the normal state was set as 100%. As seen in Additional file 3: Figure S3, where chlorpromazine and amiloride significantly reduced the uptake rate of JNP by Caco-2 cells, it indicates that the endocytosis pathway is involved in the mediation of specific reticulin and pinocytosis. However, genistein and nocodazole did not significantly inhibit the cellular uptake of JNP in either group, indicating that neither caveolin-mediated nor endocytosis was involved in the internalization of JNP.

We used a Caco2-HT29/MTX-Raji co-culture model to simulate intestinal epithelial cells, mucus-secreting cells, and M cells and evaluate the role of JNP in overcoming the intestinal epithelial cell barrier [26, 27]. In the Caco-2 cell model, the free FITC-αCT and JNP-encapsulated FITC-αCT transport volumes were lower (approximately 3% and 10% at 4 h, respectively), while the free Res transport rate was faster. There was no significant difference between free Res and JNP-encapsulated Res at 4 h (approximately 14% and 15% at 4 h, respectively). It was speculated that Res has an advantage in the osmotic transport of gastrointestinal epithelial cells due to its lipophilicity. Compared with Caco-2 cells, the presence of a mucus layer in the Caco2-HT29/MTX cell model weakened the transport of free FITC-αCT (approximately 4% at 4 h), free Res (approximately 6% at 4 h), and JNP (approximately 7% at 4 h). However, compared with the other two cell models, cumulative transport of free FITC-αCT (approximately 4% at 4 h), free Res (approximately 14% at 4 h), and JNP (approximately 13–17% at 4 h) increased in the Caco2-HT29/MTX-Raji cell model. These results suggest that the combined effects of epithelial cells, the mucus layer, and M cells contribute to the improved transport of JNP.

### Intestinal absorption and enterocytic uptake

**Fig. 6:**
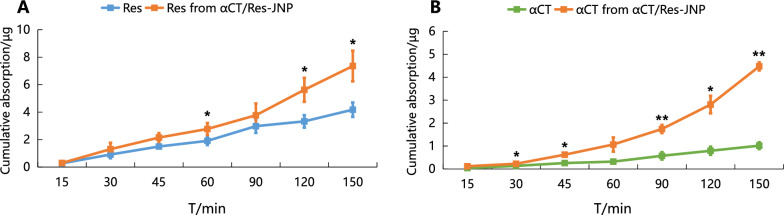
Cumulative absorption of **A** Res (*: Res *vs.* αCT/Res-JNP, *p* < 0.05) and **B** αCT (*: αCT *vs.* αCT/Res-JNP, *p* < 0.05; **: αCT *vs.* αCT/Res-JNP, *p* < 0.01) in small intestine (n = 3, mean ± SD)

Intestinal absorption and enterocytic uptake studies provided both quantitative and imaging evidence on oral absorption of αCT/Res-JNP. As shown in Fig. 6, the absorption of Res increased rapidly with time in the αCT/Res-JNP group, while it was relatively slow in the Res group. The cumulative absorption of Res in the αCT/Res-JNP group was higher than that in the Res group at all time points, and there were significant differences between the two groups at 15, 45, 60, and 90 min (p < 0.01), suggesting that JNP can promote Res absorption to a certain extent, although Res itself has good intestinal absorption characteristics. Similarly, the absorption concentration of αCT in the αCT and αCT/Res-JNP group also increased with time, but the rate of increase of the absorption concentration in the αCT group was slower than that in the αCT/Res-JNP group. The cumulative absorption of αCT in the αCT/Res-JNP group was higher than that in the αCT group at all time points, and there was a significant difference in the absorption of αCT between the αCT and αCT/Res-JNP groups at all time points except at 15 min and 60 min (p < 0.05 and p < 0.01, respectively).

We further used the rat single-pass intestinal perfusion (SPIP) model to determine the absorption mechanism of the two drugs in each intestinal segment. In SPIP, the drugs are gradually absorbed through the intestinal epithelium. It can be seen from Table 3, compared with the Res group, the absorption parameter (K_a_) and effective permeability (P_eff_) of Res in each intestinal segment in rats of the αCT/Res-JNP group were increased (K_a_ increased 1.13–1.60-fold, P_eff_ increased 1.14–1.66-fold), but there was no significant difference in the other intestinal segments except in the duodenum. The reason for this may be that the ileum is rich in drug transporters and the expression of metabolic enzymes is very low, so it is the best absorption site for Res; the absorption of αCT in the rat intestine has not been previously reported. The results also showed that there was no significant difference in the absorption of FITC-αCT in each segment of the small intestine (p > 0.05), but the absorption of FITC-αCT in the jejunum was slightly better than that in other segments. Compared with the αCT group, the K_a_ and P_eff_ of αCT in the different intestinal segments of rats in the αCT/Res-JNP group were significantly increased (K_a_ increased 1.80–2.76-fold, P_eff_ increased 1.88–3.09-fold; p < 0.01 and p < 0.05, respectively). Regardless of the transport mechanism, drugs with P_eff_ > 1.5 × 10^–4^ cm·s^−1^ in the human jejunum are considered to be completely absorbed [28], and the rat SPIP experiment provides a theoretical basis for this judgment. The absorption trends of αCT and Res from αCT/Res-JNP in different intestinal segments were the same: ileum > jejunum > colon > duodenum.

**Table 3:** Absorption of perfusate in different intestinal segments (n = 3, mean ± SD)

Intestinal segments	*Ka*/ × 10^–2^ (min^−1^)		*P*_*eff*_/ × 10^–3^ (cm·min^−1^)		*Ka*/ × 10^–2^ (min^−1^)		*P*_*eff*_/ × 10^–3^ (cm·min^−1^)	
	Res	αCT/Res-JNP	Res	αCT/Res-JNP	αCT	αCT/Res-JNP	αCT	αCT/Res-JNP
Ileum	1.91 ± 0.66	2.16 ± 0.29	3.26 ± 1.26	3.72 ± 0.57	0.91 ± 0.03	2.52 ± 0.16^∆∆^	1.44 ± 0.05	4.45 ± 0.33^∆∆^
Jejunum	1.55 ± 0.26	1.77 ± 0.17	2.57 ± 0.46	2.96 ± 0.32	0.98 ± 0.05	2.07 ± 0.43^∆^	1.56 ± 0.10	3.55 ± 0.85^∆^
Duodenum	0.99 ± 0.11	1.54 ± 0.13^**^	1.58 ± 0.18	2.54 ± 0.23^**^	0.81 ± 0.11	1.52 ± 0.37^∆^	1.27 ± 0.19	2.51 ± 0.68^∆^
Colon	1.01 ± 0.26	1.62 ± 0.45	1.60 ± 0.44	2.66 ± 0.81	0.85 ± 0.14	1.53 ± 0.42	1.34 ± 0.23	2.53 ± 0.76

Compared with free Res, ^**^ p < 0.01;

Compared with free αCT, ^∆^ p < 0.05, ^∆∆^ p < 0.01

**Fig. 7:**
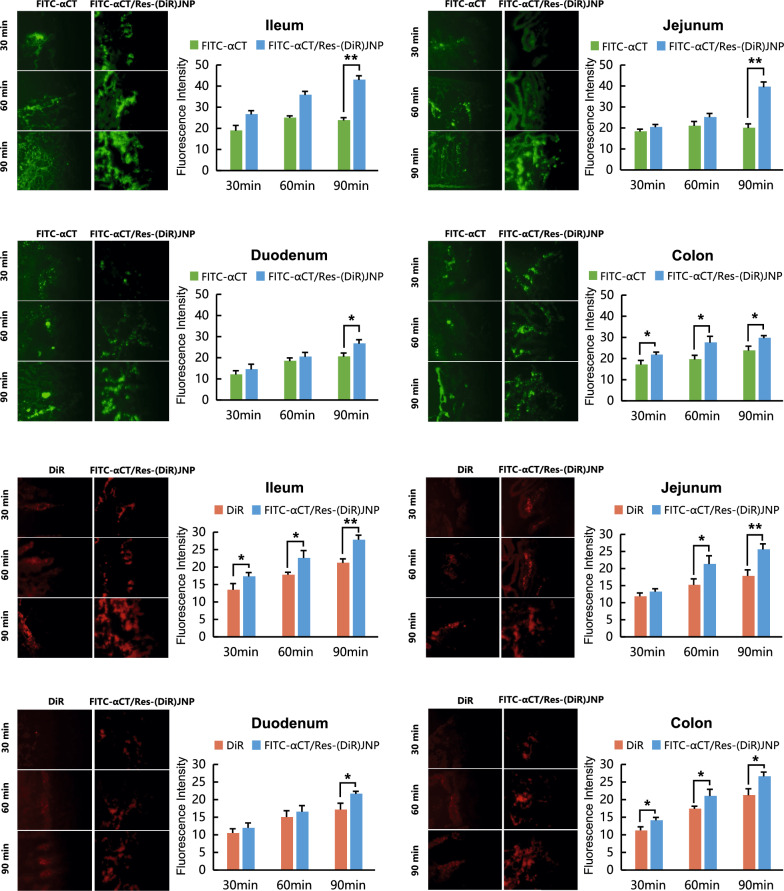
Confocal microscopic images and fluorescence intensity of the absorption of αCT/Res-JNP from different intestinal segments in the SPIP model (n = 3, mean ± SD)

The distribution and fluorescence intensity of FITC-αCT and intact JNP, which was labeled with DiR (FITC-αCT/Res-(DiR)JNP), in the different intestinal segments were observed using the SPIP model. Confocal laser scanning microscopy (CLSM) was used to observe the sections of each intestinal segment, all of which showed fluorescence, as seen in Fig. 7. Over time, the fluorescence intensity of FITC-αCT in the different intestinal segments of rats gradually increased: the fluorescence intensity of FITC-αCT in the different intestinal segments of rats in the FITC-αCT/Res-(DiR)JNP group was stronger than that in the FITC-αCT group. To further investigate the absorption of intact JNP in the small intestine, we continued to observe their absorption through intestinal epithelial cells. Since DiR is a hydrophobic dye, it is prone to fluorescence quenching once exposed to hydrophilic environment, so it was encapsulated into the oil phase of JNP as a fluorescent probe. It can be seen from Fig. 7 that with the passage of time, the fluorescence intensity of the DiR group did not increase significantly, while the fluorescence intensity of DiR in the FITC-αCT/Res-(DiR)JNP group in the different intestinal segments of rats gradually increased and was stronger than that in the control group, which indicated that JNP remained intact and was completely absorbed in each intestinal segment. The in situ SPIP study in rats confirmed that JNP was superior to the original drug in intestinal absorption capacity, which corresponded with the PK results, suggesting that JNPs have the potential to improve the bioavailability of drugs.

### Lymphatic transport

**Fig. 8:**
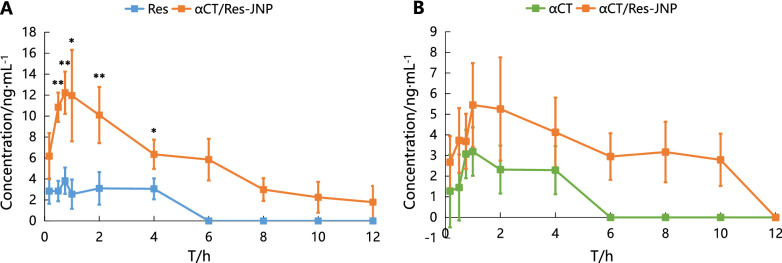
The absorption of intestinal lymphatic system at different time points after intragastric administration (n = 5, mean ± SD). **A** The intestinal lymphatic system absorption of Res (^*^: Res *vs.* αCT/Res-JNP, p < 0.05; ^**^: Res *vs.* αCT/Res-JNP, p < 0.01); **B** The intestinal lymphatic system absorption of αCT

Most oral drugs are absorbed by diffusion into intestinal epithelial cells and eventually into the bloodstream through the portal vein, while only a small amount of drugs entering epithelial cells can bind with lipoproteins and enter the bloodstream through the intestinal lymphatic system. Here, mesenteric lymph-cannulated rats were utilized to investigate the role of lymphatic transport in oral absorption of αCT/Res-JNP. From Fig. 8, it can be seen that within 12 h, both the free drug group and the JNP group had very limited lymphatic transport, and there were large differences among individuals. The concentrations of αCT and Res from αCT/Res-JNP in lymphatic transport were only about 8.72% and 6.08% of their blood concentrations at 1 h, respectively. αCT was no longer detected in the αCT group after 6 h, and there was no significant difference between the αCT and αCT/Res-JNP groups at each time point within the first 6 h (p > 0.05). Similarly, Res was no longer detected in the Res group after 4 h, but there was a significant difference between the Res group and the αCT/Res-JNP group at each time point (0.5–4 h, p < 0.05 or p < 0.01).

## Discussion

This paper provides an alternative route of administration for protein and peptide therapeutics, which usually require parenteral administration, and can be a challenge to medication adherence. The convenience and minimal pain of oral drug delivery make this route particularly attractive for chronic treatment, such as chronic pain. However, the substantial challenges of oral biologic delivery is evident. Here, Janus nanoparticles were used to realize the oral administration of macromolecular polypeptide. The co-loading capability of JNP simultaneously solves the limitation of low oral bioavailability of Res.

In the preparation process, the ethylacetate solution containing PLGA and non-ionic emulsifier PATO formed an oil phase through the surfactant, and the phase separation between the solution and the water phase was realized from the evaporation of PVA to obtain two-phase separated droplets. In the case of PVA combined with SDBS as a surfactant, with an increase in surfactant concentration, the water phase and oil phase from the phase separation changed from a core–shell structure (Fig. 1A) to a Janus structure (Fig. 1B). A higher concentration of surfactant can stabilize the interface more effectively, which is helpful in reducing the difference in surface tension between the oil and water phases [29, 30]. The JNP prepared by this method have relatively small particle size, which will affect its absorption pathway in the gastrointestinal tract. In general, nanoparticles larger than 500 nm can be phagocytosed by the microfold cells (M cells) of the collecting lymph nodes (PP) in the ileum. When the particle size is less than 500 nm, the particles can be absorbed by the intestinal epithelial cells and reach the circulatory system; [31, 32] smaller particles have an increased specific surface area and biofilm adhesion.

The release profiles observed from other PLGA nanoparticles of protein drugs is similar to that in this study, that is, it is difficult to achieve 100% cumulative release, which is due to the released free αCT may undergo a chemical reaction with PLGA, resulting in a part of αCT not being released. In the vitro long-term stability study, the aggregation between nanoparticles or hydrolytic erosion began in D5. In the presence of amino compounds, the structure of PLGA nanoparticles accelerate erosion. The presence of amino groups in LMWC may further promote the erosion of JNP, which is a possible factor affecting the aggregation of water molecules and the accumulation of osmotic pressure in the nanoparticles. Subsequent experiments would be performed in the stable period of JNP, and the form of freeze-dried powder can prolong its stable duration.

Although Res has a high oral absorption rate, it is rapidly metabolized. It is mainly converted into its sulfonic and glucuronic acid conjugates [33]. According to its concentration–time curve, a second peak appeared at 2 h, suggesting that Res was involved in enterohepatic circulation. At this time, most of the secreted bile acids are reabsorbed from the intestine and returned to the liver through portal vein circulation, thus completing the enterohepatic circulation of Res and its metabolites. After intragastric administration of Res in the form of JNP, T_max_ was delayed via endocytosis and reached the peak time at approximately 45 min. Its C_max_ was higher than that in Res group, which also suggested that JNP entrapment caused more Res to avoid the rapid liver first-pass effect. αCT was also rapidly absorbed and reached its peak concentration in about 30 min. However, it has been reported that the drug effect of αCT is delayed compared with the PK parameters of rapid uptake, and that αCT has a strong affinity to tissues with high blood perfusion (such as the kidneys, liver, lungs, heart, etc.) [34], indicating that there is a slow balance between the central and peripheral ventricles.

According to the dosage of αCT and Res after administration in the target site (PAG injection or intrathecal injection) in rats, the dosage ratio of αCT and Res was set based on the results of several reports [8, 35], which was approximately 1:5. Ban et al. found that low molecular weight hydrolysates with molecular weights less than 3000 kDa were obtained via ultrafiltration after hydrolysis of αCT in gastric juice. After intraperitoneal injection, the hydrolysate showed an obvious analgesic effect from the hot plate test in mice and the hoarseness test in rats. The effect was fast, and the pain threshold could be maintained for more than 24 h, with the effect proportional to the dose administered. Therefore, free αCT can exert an analgesic effect after oral administration. However, αCT is very sensitive to trypsin, and its analgesic effect disappears after trypsin treatment [36]. Compared with αCT/Res-JNP, free αCT and free Res only showed a weak effect on reversing mechanical hyperalgesia from D4 to D14 after operation, which may be due to the rapid decomposition and inactivation of the αCT solution in the gastrointestinal tract and the analgesic effect of free Res was limited after intragastric administration for 2 h. These results suggest that αCT and Res exert synergistic analgesic effects when they are simultaneously encapsulated in JNP. At present, αCT is considered a cholinergic receptor antagonist [35], while Res may indirectly relieve pain by balancing the release of inflammation-related cytokines [37]. The specific analgesic mechanism of their combination needs to be studied further.

From the results of intestinal absorption, the limited intestinal absorption of free αCT may be related to the hydrolysis of αCT to a certain extent, and intestinal capsules in vitro can release intestinal enzyme peptides rapidly during culture, which may also affect αCT. The protection of JNPs reduces the effects of αCT mentioned above. Due to the entrapment of JNP, the absorption trend of αCT and Res in different intestinal segments is consistent, that is, the absorption in ileum and jejunum is more than that in duodenum and colon. The reason for this may be that the duodenum degrades a lot of free drugs due to the existence of a large number of digestive enzymes, while the colon has more tight junctions and low absorption due to its special structure. On the one hand, the transmembrane transport of JNP is attributed to the bioadhesive ability of chitosan in the carrier material, which makes drugs adhere on the mucus layer rather than the intestinal cavity, shortens the absorption distance, and reduces the degradation of drugs. On the other hand, the Peyer patch surface is covered with a layer of M cells, which have advantages in absorbing biodegradable nanoparticles [38, 39]. In the absorption pathways of nanoparticles, those of the appropriate size preferentially and rapidly passed through the Peyer patch, while there are more M cells in the ileum, which may be one of the reasons why the ileum is the best absorption site.

The results of PK evaluation make it reasonable to speculate that JNPs are absorbed into the circulatory system, indicating that they may successfully cross the intestinal epithelial cells into the blood. Based on the results of cellular uptake, Res is mainly transported through intestinal epithelial cells. In its particle size range, JNPs have advantages over free drugs (especially the peptide drug αCT) in temporarily opening tight junctions and promoting cell transport. However, owing to the low content of LMWC, this effect may be limited. M cells play an important role in the transport of JNPs. However, in view of the abundance of intestinal epithelial cells and a limited number of M cells, more evidence is needed to confirm which cells (M cells or intestinal intercellular cells) are dominant in this process. In addition, the bioadhesive effect and the protective effect of JNPs can enhance the retention time of drugs in intestinal epithelial cells, promoting drug absorption and bioavailability through slow release and penetration [40, 41].

Lymphatic circulation is another transport pathway of drugs after oral administration without liver metabolism, which can reduce damage from the first-pass effect and improve oral bioavailability. The extent of drug lymphatic transport is often associated with the carrier structure and drug lipophilicity. In general, lymphatic absorption in the form of JNPs has certain advantages in improving the oral bioavailability of Res, but it was relatively limited, while the improvement in oral bioavailability of αCT was negligible. In the limited lymphatic transport of αCT/Res-JNP, part of it may be attributed to the small particle size range of αCT/Res-JNP, which was conducive to lymphatic transport. In addition, combined with the results of cell transport experiments, the lymphatic transport of αCT/Res-JNP may originate from the M cell pathway, cell bypass, and trans-cell pathways.

## Conclusions

In this study, αCT/Res-JNP were prepared and their absorption in vivo was investigated. We prepared JNP that can be loaded with two kinds of drugs with different properties and having pH-sensitive drug release characteristics in the gastrointestinal environment. From the results of the in vivo cell uptake experiments combined with fluorescence imaging of intestinal absorption and PK behavior, αCT/Res-JNP could enter systemic circulation completely through intestinal epithelial cells, which contributed to improving the oral bioavailability of the drug. In this process, the main reason for improving bioavailability is that the Peyer's patches pass through the biological mucosa, while the total absorption of lymphatic transport is very small. In conclusion, dual drug-loaded JNPs with pH sensitivity provide a new alternative for drugs that are difficult to deliver through oral administration.

## Methods

### Materials

Cobra neurotoxin (αCT, purity > 95%) was purchased from Longfenggu Biopharmaceutical Co., Ltd. (Kunming, China). Resveratrol, trimethylol aminomethane (Tris), sodium alginate (ALG), poly(ethylene lactide) (PLGA), glyceryl bisstearate (PATO), sodium dodecylbenzene sulfonate (SDBS), polyvinyl alcohol (PVA), and fluorescein isothiocyanate (FITC) were purchased from Sigma-Aldrich (St. Louis, MO, USA). Fetal bovine serum (FBS), McCoy's 5A medium, Dulbecco's modified Eagle medium (DMEM), Hank’s balanced salt solution (HBSS), and trypsin were purchased from Gibco Invitrogen Co. (Carlsbad, USA). 1,10-Dioctadecyl-3,3,30,30-tetramethylindotricarbocyanine iodide (DiR) was purchased from Aladdin Biochemical Technology Co., Ltd. (Shanghai, China). All other reagents were of analytical grade.

Male Sprague–Dawley (SD) rats weighing 200–250 g, unless otherwise specified, were purchased from Xipuer-Bikai Experimental Animal Co., Ltd. (Shanghai, China).

### Preparation of αCT/Res-JNP

Five hundred microliters (1 mg/mL) αCT was dissolved in a 2 mL HCl solution (0.1 mol/L), adjusting the pH value to 8.0–8.4 using Tris. The solution was dissolved in CaCl_2_ (0.5 mmol/L), and added dropwise to ALG (2 mg/mL). The solution was then ultrasonicated for 15 min. Approximately 10 mL of LMWC (2 mg/mL) was prepared, adjusting the pH to 6.5 using NaOH (0.1 M). While stirring, normal saline solution was added to the LMWC solution, adding in ALG, and then continuously stirred for 1 h to form an internal water phase. The oil phase was composed of PLGA and precision ATO5 in a certain proportion (75:25). It was then dissolved in ethyl acetate, in which 2.5 mg Res and span 80 were added. The internal water and oil phases formed a W1/O emulsion under ultrasonic emulsification. Then, a surfactant (PVA:SDBS = 1:3) was added to the W1/O emulsion, and the W1/O/W2 double emulsion was formed via ultrasonic emulsification. The ethyl acetate solution was removed by stirring the solution at 40 °C for 4 h to form JNPs. During the evaporation process, LMWC/ALG was separated from the lipids to form anisotropic Janus particles. The suspension was centrifuged three times and washed with deionized water to remove residual free drug and surfactant. JNP-loaded αCT (αCT-JNP) or Res (Res-JNP) were prepared using the same method.

### Preparation of fluorescent labeled αCT/Res-JNP

For fluorescence observation, FITC or DiR were used to label the αCT and oil phases, respectively. We used the previous technology and research basis of our group to prepare FITC-αCT via HPLC [42]. According to different investigation purposes, following JNPs were prepared with fluorescent markers: (1) FITC-αCT-JNP: only αCT loaded JNP using FITC-αCT instead of αCT; (2) FITC-αCT/Res-JNP: dual-drug loaded JNP using FITC-αCT instead of αCT; (3) FITC-αCT/Res-(DiR)JNP: dual-drug loaded JNP using FITC-αCT instead of αCT and DiR was mixed in ethyl acetate [43]. The other preparation method is the same as the preparation of αCT/Res-JNP.

### Characterization of JNPs

The morphology of αCT/Res-JNP was observed using transmission electron microscopy (TEM) (JEM-1200EX, JEOL, Japan), and the average particle size, particle size distribution, and zeta potential were measured using a particle size potential analyzer (Zetasizer Nano ZS, Malvern, UK). The structure of αCT/Res-JNP was detected by XRD (XRD-7000, Shimadzu, Japan) (Additional file 4: S4). Ultracentrifugation was performed for the αCT/Res-JNP suspension, and the supernatant was filtered through a 0.22-μm microporous membrane. The concentration of free αCT and Res in the filtrate was determined via HPLC, which was calculated as C1; the initial concentration of αCT and Res was calculated as C0 according to the dosage; and the concentration of αCT/Res JNP was calculated as M (the mass ratio of lyophilized αCT/Res-JNP to the volume ratio of colloidal solution). The formulae for EE and DL is as follows:

1 $${\text{EE}}\% = \left( {{\text{C}}0 \, - {\text{ C1}}} \right)/{\text{C}}0 \times {1}00\%$$

2 $${\text{DL}}\% = \left( {{\text{C}}0 \, - {\text{ C1}}} \right)/{\text{M}} \times {1}00\%$$

### *Release behavior *in vitro

The release behavior of αCT and Res in vitro was investigated through dialysis. Two milliliters of Res, αCT, αCT-JNP suspension, Res-JNP suspension, and αCT/Res-JNP suspension were precisely measured and placed in a dialysis bag (Mw = 12 kDa-14 kDa). The release medium was SGJ (pH 1.2) containing 2% sodium dodecyl sulfate (w/v) and 30 mL PBS (pH 7.4). The release behavior of drugs from JNP was studied under the conditions of 37 ± 0.5 °C and a rotating speed of 100 rpm. At fixed time points (0, 0.25, 0.5, 0.75, 1.0, 1.5, and 2.0 h in SGJ; 0, 0.25, 0.5, 1.0, 2,0, 4.0, 6.0, 8.0, 10.0, 12.0, and 24.0 h in PBS), 1 mL of release medium was removed, and a blank release medium with the same volume and temperature was supplemented. The samples were filtered through a 0.22-μm microporous membrane, and the supernatant was obtained after high-speed centrifugation. The αCT and Res content were determined using HPLC. The cumulative release percentage was calculated, and a release curve was obtained. The formula for cumulative drug release is shown in the following equation, where C_t_ is the content of two drugs at each sampling point, V_0_ is the volume of the release medium, V is the sampling volume, and M_0_ is the initial dosage:

3 $${\text{Cumulative release}}\% = \left( {C_{t} \times V_{0} + \sum\nolimits_{n = 1}^{t - 1} {C \times V} } \right)/M_{0} \times 100\%$$

### In vitro* stability*

The short-term and long-term in vitro stability were investigated in SIF (pH 6.8) and PBS (pH 7.4), respectively. Using SIF (pH 6.8) as the dissolution medium, drug-loaded JNP and blank JNP suspensions were cultured in SIF at 37 °C for 12 h. The average particle size and zeta potential were determined immediately after the samples were extracted at predetermined time points (0, 2, 4, 6, 8, 10, 12 h). Similarly, the average diameter and zeta potential of the two groups of JNP from D1 to D5 were determined by the same method with PBS (pH 7.4) as the dissolution medium.

The effect of trypsin on the stability of αCT/Res-JNPs was also evaluated. αCT/Res-JNPs were centrifuged at 20,000 rpm at 4 °C for 30 min, and then resuspended in SIF. The control group was treated with αCT solution and Res solution. SIFs and trypsin (0.2%, w/v) were mixed at 37 °C for 2 h. At predetermined time points (15, 30, 60, and 120 min), HCl solution (0.1 M) was added to terminate the trypsin activity. Subsequently, dichloromethane was added to dissolve the organic phase contained in the JNP solution. The pH of the solution was then adjusted to 4.0 using hydrochloric acid to dissolve the internal aqueous phase of the JNP. Subsequently, samples were collected, and the contents of residual αCT and Res in the JNP samples were analyzed via HPLC.

### Pharmacokinetics

The pharmacokinetic (PK) study was divided into five groups: αCT/Res-JNP, αCT solution combined with Res-JNP, Res solution combined with αCT-JNP, and Res solution combined with αCT solution (the αCT in all groups were labeled with FITC). SD rats fasted but drank water freely for 24 h before administration. The dosage of JNP group was 50 µg·kg ^−1^ (calculated using αCT), and the other groups were converted according to the ratio of their drug loading to αCT. After intragastric administration, blood samples were collected at 0.25, 0.5, 1, 4, 8, 12, and 24 h. The contents of FITC-αCT and Res were determined via capillary electrophoresis coupled with laser-induced fluorescence detection (CE-LIF, AB Sciex, USA) and UPLC-QTOF-MS/MS (Sciex X-500R, Sciex, USA), respectively. PKSolver software (V2.0, China Pharmaceutical University, China) was used to process the data, compare the blood PK parameters of each group after administration, and generate the drug concentration–time curve.

### Analgesic effect

The analgesic effects of αCT/Res-JNP in normal SD rats and SCI model rats were determined using the hot plate test (acute pain model) and SCI model (central pain model). In the hot plate test, the temperature of the hot plate instrument was adjusted to 55 ± 0.5 °C, and rats (female, 200–250 g) were placed on a hot plate. Forty rats with normal pain response times of 5–30 s were randomly divided into four groups: NS control, αCT solution combined with Res solution, Res solution combined with αCT-JNP, and αCT/Res-JNP. The dosage was the same as the pharmacokinetic study. After intragastric administration, the pain response time was measured at 15, 30, 45, 60, 90, 120, 180, 240, 300, 360, 420, and 480 min. If there was no pain reaction after 60 s, the rats were removed immediately to avoid scalding. The pain reaction time was calculated to be 60 s. Taking maximal possible effect (%MPE) as the evaluation index, the pharmacodynamics of each preparation were determined by comparing the value of %MPE. The calculation formula is as follows, in which the cut-off time was recorded as 60 s:

4 $$\% {\text{MPE}} = \left( {{\text{post}} - {\text{drug latency }}{-}{\text{ pre}} - {\text{drug latency}}} \right)*{1}00/\left( {{\text{cut off time }}{-}{\text{ pre}} - {\text{drug latency}}} \right)$$

The analgesic experiment was performed using the SCI model, which was made according to previous reports [44]. A total of 50 SD rats (male, 200–250 g) were randomly divided into five groups: 1) normal control group: rats were given normal saline every day without operation; 2) sham-operated group: rats were anesthetized, exposing only the vertebral lamina after skin incision without spinal cord injury; 3) SCI model group: rats were given normal saline every day after operation; 4) SCI model + αCT/Res-JNP solution group: rats were given SCI operation modeling and αCT/Res-JNP every day after operation; and 5) SCI model + free drug group: rats were given SCI operation modeling and free αCT and Res solution every day after operation. The dosage was 50 µg·kg ^−1^ (calculated using αCT) for 28 days. According to the hot plate method, the best analgesic time was predicted to be 2 h after intragastric administration. The von Frey filament was used to measure the mechanical foot retraction reflex threshold through the “up and down” method [45] before operation (without administration) and at 2 h after intragastric administration on days 4, 7, 10, 14, 21, and 28 post-operation. The median method was used to calculate the 50% threshold of foot retraction, according to the following equation:

5 $${5}0\% {\text{ foot retraction threshold }}\left( {\text{g}} \right) = \left( {{1}0^{{[{\text{Xf}} + {\text{k}}\delta ]}} } \right)/{1}0,000$$

where f is the strength of the last filament, XF is log (f*10,000); δ is the average difference of the strength of each filament after taking the logarithm, which is about 0.224; k is the value obtained by looking up the table according to the measured "X" and "O" sequences. [46]

### Cellular uptake and trans-monolayer transport

Cell culture was carried out according to previously described procedures. HT-29/MTX and Caco-2 cells were seeded into 24-well plates at a density of 1 × 10^5^ cells/well and 6 × 10^4^ cells/well, respectively. After the cells adhered onto each well, the culture medium was discarded, and three types of samples (FITC-αCT, FITC-αCT-JNP, or FITC-αCT/Res-JNP) at 1:20 dilutions were added. After 6 h, the liquid in the wells was discarded, and the cells were washed with PBS. Each well was fixed with 4% (v/v) paraformaldehyde (0.5 mL) for 20 min, and rinsed with PBS. The cells were then incubated with 0.1% Triton X-100 for 10 min to permeabilize the cell membrane, and then incubated with DAPI for 10 min. After sealing, it was observed using CLSM.

The mechanism of cell uptake was also investigated. Caco-2 cells in the logarithmic growth phase were seeded in 6-well plates at 3 × 10^5^ cells/well and incubated for 48 h. Chlorpromazine (10 μg/mL), genistein (54 μg/mL), amiloride (12 μg/mL), and nocodazole (300 μg/mL) were added to the individual wells as inhibitors and were incubated for 0.5 h. FITC-αCT-JNP and FITC-αCT/Res-JNP were then added and the wells were incubated for another 2 h. The cells were then collected using trypsin and centrifuged at 1000 rpm for 5 min. Cells without any inhibitors were used as controls. Fluorescence intensity was detected using flow cytometry (FACSJazz, BD, USA).

Caco-2, Caco-2/HT29-MTX (7/3), and Caco-2/HT29-MTX/Raji cell models were established for the trans-monolayer transport study, as previously described [47]. The assay was carried out from the AP side (pH 6.5) to the BL side (pH 7.4) using HBSS-HEPES buffer. Free FITC-αCT (3 μg/mL), free Res (50 μg/mL), and FITC-αCT/Res-JNP were added to the top of the chamber. At 0, 0.5, 1, 2, and 4 h, a certain amount of sample was taken from the basolateral compartment, and the same volume of HBSS-HEPES buffer was added. The fluorescence intensity was measured at 488 nm using a microplate reader, and the Res concentration was determined using UPLC.

### Intestinal absorption and enterocytic uptake

The in vitro intestinal absorption of FITC-αCT and Res was determined through eversion absorption in the intestinal sac. At 15, 30, 45, 60, 90, 120, and 150 min, 0.1 mL intestinal sac solution was taken for testing and supplemented with an equal volume of blank Krebs-Ringe (K-R) nutrient solution. The concentrations of FITC-αCT and Res in the K-R nutrient solution were determined using CE-LIF and UPLC-Q/TOF, respectively. The drug concentration and cumulative absorption (Q) were calculated.

SPIP was performed to determine the absorption and enterocytic uptake of the nanoparticles. The abdominal cavity of SD rats were opened along the midline of the abdomen to expose the intestinal tissue. The jejunum, ileum, duodenum, and colon segments were measured, and incisions were made at both ends of these segments. Both ends of the incision were intubated and fixed using ligation. The wounds were covered with an absorbent cotton soaked in normal saline and maintained at 37 °C. When the perfusion fluid (free αCT, DiR, FITC-αCT/Res-(DiR)JNP) filled the whole circulation pathway, it was recorded as time 0, and the fluorescence intensity related to the intestinal segment was detected using a real-time imaging system at 0, 30, and 60 min. After 60 min, the jejunum, ileum, duodenum, and colon were dehydrated to create frozen Sects. (10 μm). These sections were observed using CLSM, and the fluorescence intensity of each section was quantified. The absorption rate constant K_a_ and effective permeability P_eff_ of FITC-αCT and Res in the small intestine were also evaluated using SPIP, and the concentration of FITC-αCT and Res in the perfusate were determined to investigate drug absorption.

### Lymphatic transport

The methods of mesenteric lymphatic intubation were based on previous studies [48]. Briefly, SD rats (300–350 g) were given 2 mL peanut oil via gavage 1 h before administration to induce lymphoid production. One hour later, the animals were anesthetized via intraperitoneal injection of 50 mg·kg^−1^ pentobarbital sodium. The rats were placed in the supine position on the ventral side and their limbs were fixed. A midline abdominal incision (approximately 4 cm long) was made at 2/3 of the line connecting the xiphoid and pubic symphysis. The small intestine was removed, and the inferior vena cava and left renal vein were exposed. Milky white mesenteric lymphatic vessels parallel to the mesenteric artery were observed at the root of the mesenteric artery. The trunk of the mesenteric lymphatic vessels (approximately 1 cm) was then separated. A 4-mm PE-25 pipe with a PE-50 hose was connected, the joint of each pipe was fully fixed, and the pipe was filled with 1% EDTA-2Na. A catheter was passed through the inferior vena cava and was parallel to the mesenteric lymphatic vessels. A small incision was made in the mesenteric lymphatic vessel, and the PE-25 tube end was intubated. When the lymphatic fluid completely filled the tube and drained smoothly, the intubation site was fixed. After resuscitation, the rats were allowed to drink 5% glucose solution freely and recover overnight. After enema administration (the dosage was the same as the pharmacokinetic study), lymph samples were collected continuously at the predetermined time points to determine the concentration of αCT and Res.

### Data analysis

Statistical analyses of mean results across multiple treatment groups were performed by Origin 6.0 (Microcal Software, Inc., USA) using ANOVA, followed by paired Student’s t-test. A P value below 0.05 was considered to indicate significant difference between means. All values were presented as mean ± standard deviation (SD).

## Supplementary Information

**Additional file 1.** Synergistic analgesic effect of αCT combined with Res.

**Additional file 2.** Cytotoxicity study.

**Additional file 3.** Effect of specific endocytosis inhibitor on the uptake of Caco-2 cells.

**Additional file 4.** Method of X-ray diffraction.

## Data Availability

All data generated or analysed during this study are included in this published article and its additional file.
