# Supplementary material for: pH-sensitive dual drug loaded janus nanoparticles by oral delivery for multimodal analgesia
Source: J Nanobiotechnology. 2021 Aug 6;19:235. doi: 10.1186/s12951-021-00974-6 (PMC8348996; doi:10.1186/s12951-021-00974-6)
Supplement: Supplementary file 1 — Additional file 1. Synergistic analgesic effect of αCT combined with Res. [file 12951_2021_974_MOESM1_ESM.docx]

**S1:** *Synergistic analgesic effect of* αCT *combined with Res*

*Preparation of αCT-NP* The procedures used for preparing the αCT-nanoparticle (αCT-NP) were double emulsification solvent evaporation method. Briefly, a 50 mL aliquot of the αCT solution containing 50 mg of toxin was emulsified in 1 mL of PLGA in ethyl acetate (40 mg/mL) by vortex for 40 s. Then, 2 mL of aqueous sodium cholate solution (1.5 %, w/v) was added and the resulting (W/O)/W emulsion was sonicated for 30 s (90 W). The double emulsion was diluted in 40 mL PVA solution (0.2 %, w/v) and the solvent was rapidly eliminated by evaporation under vacuum. The nano-colloidal solution of αCT-loaded-NPs were concentrated and collected.

*Preparation of Res-NP* A certain amount of Res (Res was dissolved in ethanol) was added to the PLGA (PLGA was dissolved in dichloromethane and acetone), and sonicated at 200 W for 2 min to produce a primary emulsion (organic phase). Then the primary emulsion was slowly injected into the BSA solution (1% w/v) (aqueous phase) and sonicated at 200 W for 4 min again. The final oil/water (O/W) emulsion was made. To disperse the final O/W emulsion, 15 ml of deionized water was added and stirred magnetically for some hours to remove the residual organic solvent. After being centrifuged at 14 000 r.p.m. for 30 min, nanoparticles were obtained and the supernatant was removed.

*Hot plate test* The analgesic effects of αCT combined with Res in normal SD rats were determined using the hot plate test. In the hot plate test, the temperature of the hot plate instrument was adjusted to 55 ± 0.5 °C, and rats (female, 200-250 g) were placed on a hot plate. Forty eight rats with normal pain response times of 5-30 s were randomly divided into four groups: (1) αCT solution, (2) Res solution, (3) αCT solution combined with Res solution, (4) αCT loaded nanoparticle (αCT-NP), (5) Res loaded nanoparticle (Res-NP), and (6) αCT-NP combined with Res-NP. The dosage of αCT and Res were 50 μg·kg^-1^ and 2 mg·kg^-1^, respectively After intragastric administration, the pain response time was measured at 15, 30, 45, 60, 90, 120, 180, 240, 300, 360, 420, and 480 min. If there was no pain reaction after 60 s, the rats were removed immediately to avoid scalding. The pain reaction time was calculated to be 60 s. Taking maximal possible effect (%MPE) as the evaluation index, the pharmacodynamics of each preparation were determined by comparing the value of %MPE. The calculation formula is as follows, in which the cut-off time was recorded as 60 s:

%MPE=(post-drug latency – pre-drug latency)*100/(cut off time – pre-drug latency)

*Analgesic effect* The results showed that the maximal analgesic effects of both αCT and Res were enhanced when they were encapsulated in nanoparticles (**Figure S1A and Figure S1B)**, which may be due to the contribution of nanoparticles to gastrointestinal absorption. As can be seen from **Figure S1C**, αCT and Res have synergistic analgesic effect after combined oral administration, that may be attributed to their multi-target role. In addition, when the two drugs are combined oral administration in the form of nanoparticles, the synergistic effect can be further enhanced (**Figure S1D**).


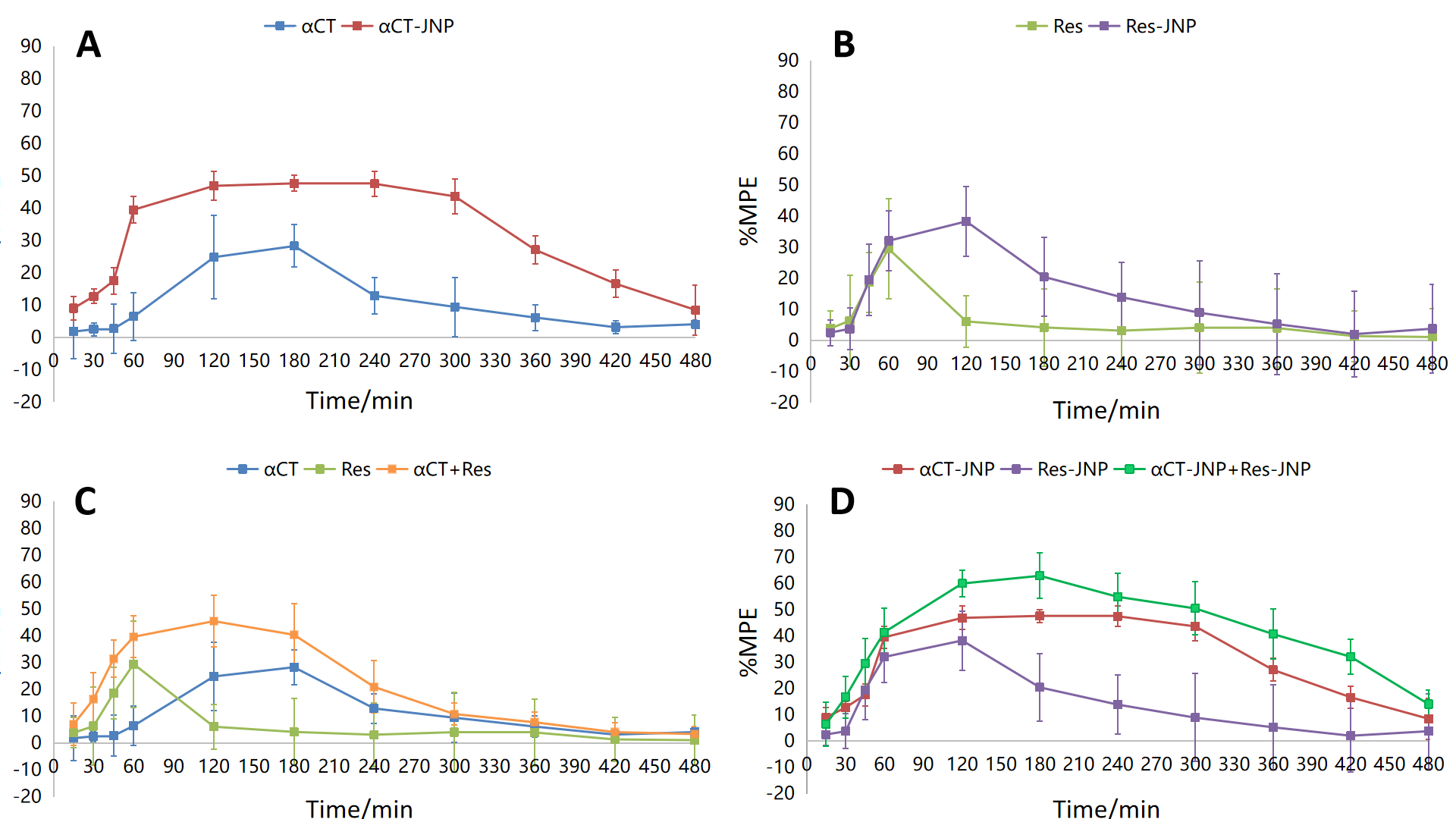


Figure S1 %MPE of hot plate test after intragastric administration in rats. (n=8, mean ± SD)
