# Supplementary material for: pH-sensitive dual drug loaded janus nanoparticles by oral delivery for multimodal analgesia
Source: J Nanobiotechnology. 2021 Aug 6;19:235. doi: 10.1186/s12951-021-00974-6 (PMC8348996; doi:10.1186/s12951-021-00974-6)
Supplement: Supplementary file 2 — Additional file 2. Cytotoxicity study. [file 12951_2021_974_MOESM2_ESM.docx]

**S2：Cytotoxicity study**

*Cell culture* Add 10 ml culture medium (HT-29/MTX cells were cultured in 88% DMEM medium containing 10% fetal bovine serum, 1% nonessential amino acids and 1% double antibody; Caco2 cells were cultured in DMEM basic medium containing 15% fetal bovine serum and 1% penicillin/streptomycin), transfer the cells to the culture dish. The cells were cultured at 37 °C and 5% CO_2_. When the cells grow into a tight monolayer in the culture dish, it means that they are close to saturation state, and the cells are subcultured. Take the normal cells in logarithmic phase, discard the original culture medium, add 1ml 0.25% trypsin, digest for about 2 min; observe under the microscope, add 2ml culture medium to stop digestion after all the cells become round and bright. The cell suspension was collected and centrifuged at 1500 rpm for 3 min. The supernatant was discarded and resuspended in 1 ml complete medium. The supernatant was added into the medium containing culture dish according to a certain proportion, and the culture was continued at 37℃ and 5% CO_2_.

*Cell viability (CCK8)* The cells in logarithmic growth phase were digested with 1 ml 0.25% trypsin, terminated with 2 ml complete medium, transferred to 15 ml centrifuge tube, centrifuged at 1500 rpm for 3 min, counted under the counting plate, and inoculated into 96 well plate according to HT-29/MTX: 3 × 10^4^ cells/well, Caco2: 1 × 10^4^ cells/well. The confluence degree of cells was about 80%, and cultured overnight at 37 ℃ in 5% CO_2_. On the next day, different concentrations of FITC-αCT-JNP and FITC-α CT/Res-JNP were added after cell adhesion. The concentrations of FITC-α CT-JNP and FITC-αCT/Res-JNP were 0%, 0.1%, 1%, 5%, 10%, 15%, 20% respectively. At the same time, the control group was set as pure culture medium (sterile water 1%, 10%, 20%). CCK8 was detected at 0 h, 24 h, 48 h and 72 h respectively. CCK8 solution (5mg/ml) was added into 96 well plate and incubated in dark for 2h. The absorbance of each well was measured at 450 nm by microplate reader (IX73, BioTek Instruments, USA).

*Cell activity* As shown in **Figure S2**, after incubation with FITC-αCT-JNP or FITC-αCT/Res-JNP in the concentration range of 0-20% (i.e. αCT is about 0-8 μg/ml, Res is about 0-350 μg/ml), the cell viability was stable when the concentration of FITC-αCT-JNP and FITC-αCT/Res-JNP was less than 10% within 72 h (Caco-2 is 78%-116%, HT-29/MTX is 80%-128%), indicating that FITC-αCT-JNP and FITC-αCT/Res-JNP have no significant effect on the cell survival rate at this concentration. However, when the drug concentration was greater than or equal to 15%, the cell activity decreased. By observing the cell state under the microscope, we found that the proliferation of the two kinds of cells were inhibited. Therefore, in the cell experiments in this paper, the concentration of FITC-αCT-JNP and FITC-αCT/Res-JNP was not exceed 10%.


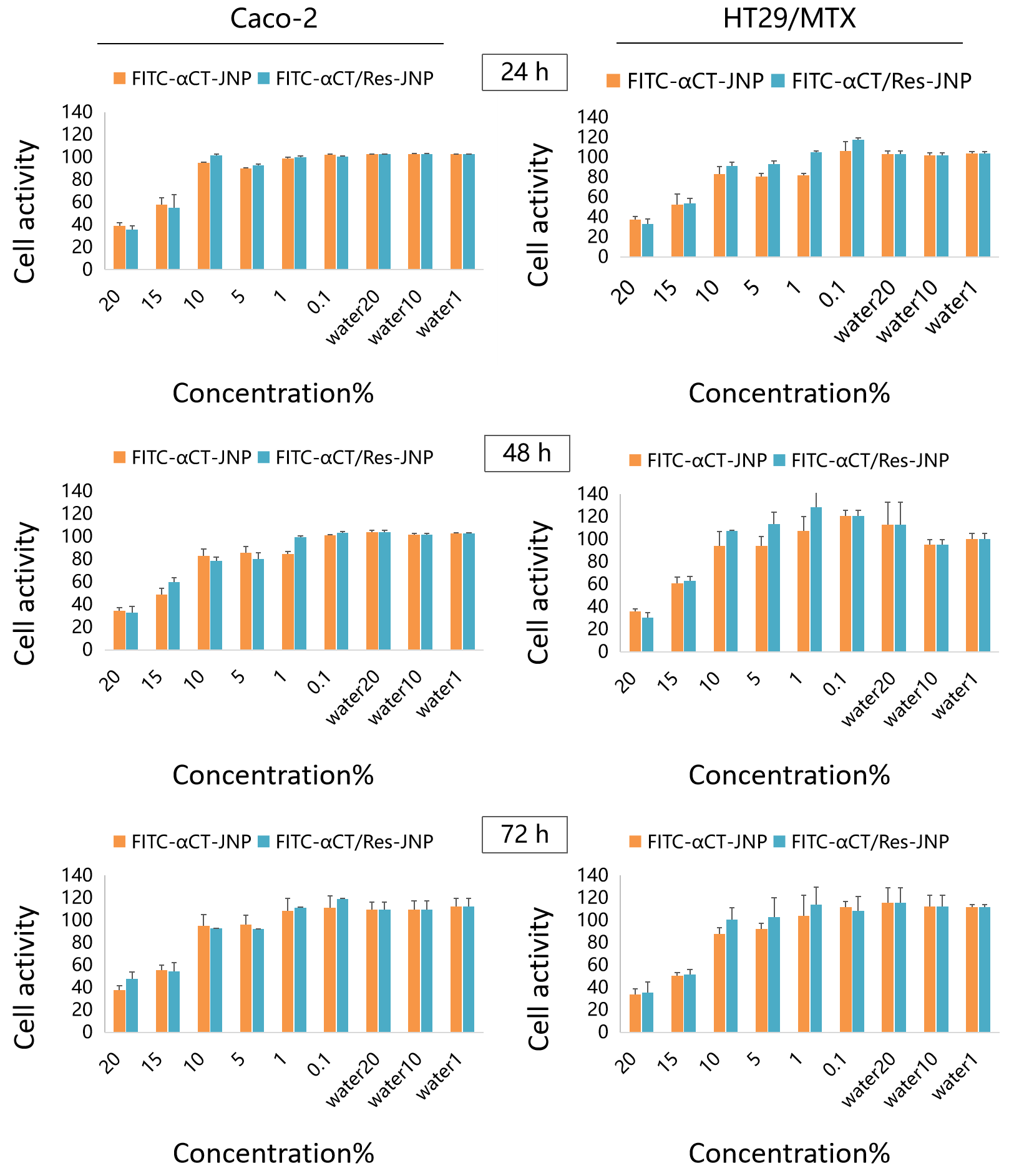


Figure S2 Cell activity of Caco-2 cells and HT29/MTX cells incubated with FITC-αCT-JNP and FITC-αCT/Res-JNP for 24 h, 48 h and 72 h, respectively
