# Supplementary material for: pH-sensitive dual drug loaded janus nanoparticles by oral delivery for multimodal analgesia
Source: J Nanobiotechnology. 2021 Aug 6;19:235. doi: 10.1186/s12951-021-00974-6 (PMC8348996; doi:10.1186/s12951-021-00974-6)
Supplement: Supplementary file 3 — Additional file 3. Effect of specific endocytosis inhibitor on the uptake of Caco-2 cells. [file 12951_2021_974_MOESM3_ESM.docx]

**S3: Effect of specific endocytosis inhibitor on the uptake of Caco-2 cells**


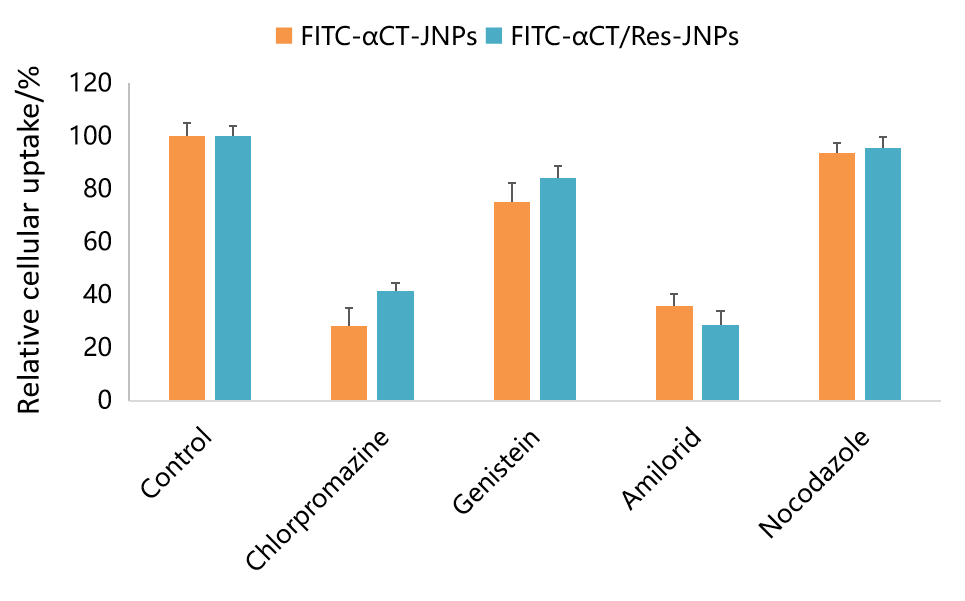


Figure S3 Relative cellular uptake of FITC-αCT-JNPs and FITC-αCT/Res-JNPs by Caco-2 cells under different endocytosis inhibitors (n = 3, mean±SD)
