# Supplementary material for: pH-sensitive dual drug loaded janus nanoparticles by oral delivery for multimodal analgesia
Source: J Nanobiotechnology. 2021 Aug 6;19:235. doi: 10.1186/s12951-021-00974-6 (PMC8348996; doi:10.1186/s12951-021-00974-6)
Supplement: Supplementary file 4 — Additional file 4. Method of X-ray diffraction. [file 12951_2021_974_MOESM4_ESM.docx]

**S4: Method of X-ray diffraction**

Samples to be tested were recorded on X-ray diffractometer (XRD-7000, Shimadzu, Japan) using Cu Kα radiation (λ=1.5406 Å), generated with 40 kV accelerating potential and 20 mA tube current with monochromatic radiation under Bragg-Brentano geometry. An XRD spectrum was acquired under ambient conditions in the angle range from 10 to 80° with a step size of 0.02° every 0.60 s. Samples were placed on nonabsorptive glass in the form of fine powder.

Scanning samples were as follows: (a) αCT, (b) Res, (c) carrier material mixture (PLGA:LWMC:ALG = 1:1:1), (d) αCT + Res + carrier material mixture (αCT:Res:PLGA:LWMC:ALG=1:1:1:1:1), (e) αCT/Res JNP lyophilized powder.
